# Detection of organic compounds in freshly ejected ice grains from Enceladus’s ocean

**DOI:** 10.1038/s41550-025-02655-y

**Published:** 2025-10-01

**Authors:** Nozair Khawaja, Frank Postberg, Thomas R. O’Sullivan, Maryse Napoleoni, Sascha Kempf, Fabian Klenner, Yasuhito Sekine, Maxwell Craddock, Jon Hillier, Jonas Simolka, Lucía Hortal Sánchez, Ralf Srama

**Affiliations:** 1 Institute of Space Systems, University of Stuttgart, Stuttgart, Germany; 2 Freie Universität Berlin, Institute of Geological Sciences, Berlin, Germany; 3 Laboratory for Atmospheric and Space Physics, University of Colorado, Boulder, CO USA; 4 Department of Earth and Space Sciences, University of Washington, Seattle, WA USA; 5 Earth-Life Science Institute (ELSI), Institute of Science, Tokyo, Japan; 6 Present Address: Freie Universität Berlin, Institute of Geological Sciences, Berlin, Germany

**Keywords:** Astrobiology, Rings and moons

## Abstract

Saturn’s moon Enceladus ejects a plume of ice grains and gases originating from a subsurface ocean via fractures near its south pole. The chemical characterization of organic material in such ice grains was previously conducted via the analysis of mass spectra obtained in Saturn’s E ring by Cassini’s Cosmic Dust Analyzer at impact speeds below 12 km s^−1^. Here we present a comprehensive chemical analysis of organic-bearing ice grains sampled directly from the plume during a Cassini fly-by of Enceladus (E5) at an encounter speed of nearly 18 km s^−1^. We again detect aryl and oxygen moieties in these fresh ice grains, as previously identified in older E-ring grains. Furthermore, the unprecedented high encounter speed revealed previously unobserved molecular fragments in Cosmic Dust Analyzer spectra, allowing the identification of aliphatic, (hetero)cyclic ester/alkenes, ethers/ethyl and, tentatively, N- and O-bearing compounds. These freshly ejected species are derived from the Enceladus subsurface, hinting at a hydrothermal origin and involvement in geochemical pathways towards the synthesis and evolution of organics.

## Abstract (web summary)

Fresh ice grains from Enceladus’s plume were analysed during the highest-speed fly-by of the Cassini spacecraft. Organic compounds with a range of chemical structures were discovered, suggesting comprehensive subsurface chemistry on Enceladus.

## Main

The Saturnian moon Enceladus emits a plume of water ice grains and volatiles through surface fractures at its south pole. The Cassini–Huygens space mission conducted compositional analysis, both in situ with its mass spectrometers—the Cosmic Dust Analyzer^1^ (CDA) and the Ion and Neutral Mass Spectrometer^2^ (INMS)—and with the Ultraviolet Imaging Spectrograph^3^, which acquired compositional data from plume observations, indicating an oceanic origin of this material^4–11^. A global, salty subsurface ocean percolates through Enceladus’s rocky core, where hydrothermal activity is thought to occur^6,12–16^. The vented material from Enceladus contains a variety of organic and inorganic species originating from the subsurface ocean^4–10,17,18^. The recent identification of phosphates^9^ in the plume means that five of the six bioessential CHNOPS elements have been detected in material from Enceladus.

Cassini’s CDA recorded hundreds of thousands of in situ time-of-flight (TOF) mass spectra of ice grains in the E ring. After ejection from Enceladus’s interior, about 10% of these grains^19^ settle across the E ring over days to decades at distances of about 2.5–20 *R*_S_ (Saturn radii *R*_s_ = 60,330 km)^20–22^. Mass spectral analysis revealed three distinct compositional types of Enceladean ice grains in the E ring^6,8,10,23^: type I, almost pure water ice; type II, organic enriched; and type III, salt rich. In type II E-ring ice grains, Khawaja et al.^10^ found volatile, low-mass (≤100 u; u = atomic mass unit), N- and O-bearing organic species as well as single-ringed aromatic compounds. In a particular type II subtype, Postberg et al.^8^ discovered complex macromolecular fragments of refractory insoluble organic compounds with masses exceeding 200 u, with multiple aryl moieties connected to chains of saturated and unsaturated hydrocarbons, alongside N- and O-bearing groups.

While the majority of previous results were inferred from relatively old E-ring ice grains, fly-bys of Enceladus by Cassini provided a unique opportunity to sample freshly ejected grains. This offers compositional insights into ice grains immediately after ejection and ensures that the compounds detected arise from the Enceladean subsurface rather than space weathering in Saturn’s E ring^22^. The impact speed of ice grains significantly influences the spectral appearance of their mass spectra^24^. While spectra of ice grains in Saturn’s E ring were mostly recorded between 4 and 12 km s^−1^, Cassini’s E5 fly-by occurred at the highest speed (17.7 km s^−1^) of all Enceladus fly-bys, offering new diagnostics for the analysis of previously unseen high-energy-induced fragmentation pathways. Simultaneously, INMS recorded measurements during the E5 fly-by, showing a similar correlation between fly-by speed and extent of fragmentation^25^. The absence of water-cluster species—which are prevalent below 12 km s^−1^ and can mask signals arising from organic species—at such high impact velocities is advantageous. Here we reanalyse the data from Cassini’s E5 fly-by to identify specific organic species within type II grains, from which Postberg et al.^7^ estimated relative proportions of ice grain types without detailed compositional analysis.

## Results

In this E5 dataset we identify certain groups of CDA mass spectra indicative of different organic compositions in freshly ejected ice grains: aromatics, O-bearing (probably carbonyl), esters/alkenes and ethers/ethyls. Each of these groups will be discussed in the following sections using archetypal spectra as illustrative examples. CDA utilized impact ionization, whereby (sub-)micrometre-sized ice grains collide with the instrument’s target at hypervelocities (≳2–3 km s^−1^), resulting in the formation of ions (and other fragments) which generate TOF mass spectra. In this study, the cation mass spectra obtained at an encounter speed of 17.7 km s^−1^ frequently exhibit features characteristic of this high impact velocity—namely [H_2,3_]^+^, [C]^+^ and [Rh]^+^ ions. These rhodium and hydrogen cations originate from the material utilized for the CDA’s impact target^26^.

**Extended Data Fig. 1:**
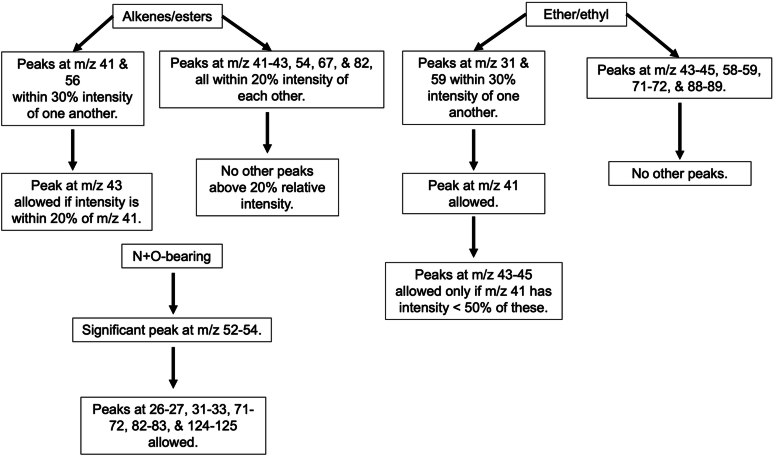
Flowchart of spectral types. A flowchart describing the selection of EI spectra for each class of organic compound based upon their EI peaks.

In this study, laboratory electron ionization (EI) mass spectra were employed to facilitate spectral interpretation in accordance with ref. ^27^. In comparison to the laser-induced liquid beam ion desorption (LILBID)^24^ that match CDA spectra at the lower spacecraft–ice grain encounter speeds obtained in the E ring, EI spectra match the observed ionic species particularly well at higher impact speeds. We selected the EI mass spectra shown in this work based upon relative peak intensities of the characteristic fragments of a given species, as described in the Methods section (see also Extended Data Fig. 1). All EI spectra in this work were obtained from the National Institute of Standards and Technology (NIST), MassBank Europe and MassBank of North America (MoNA) freely available online databases. It should be noted that all EI spectra from these databases correspond to pure organic species. In contrast to the CDA spectra, they do not show any species from water ice nor traces of Na and K salts that are ubiquitous in type II spectra. The proposed processes^8,10^ of the formation of organic-enriched ice grains suggest that each ice grain could contain more than one type of organic species. While contributions from more than one type of organic may also complicate identification of chemical species, in general, the defining features of organics in ice grain mass spectra must belong to those compounds with a lower ionization potential, or to those dominating the ice grain composition in the case of multiple compounds with similar ionization potentials.

If present, mass spectral features at mass-to-charge (*m*/*z*) ratios of 12–15 (C, CH^+^, ${{\rm{CH}}}_{2}^{+}$, ${{\rm{CH}}}_{3}^{+}$), 16–19 (O^+^, HO^+^, H_2_O^+^, H_3_O^+^), 23 (Na^+^), 27–29 (${{\rm{C}}}_{2}{{\rm{H}}}_{3}^{+}$, ${{\rm{CH}}}_{4}^{+}$, ${{\rm{C}}}_{2}{{\rm{H}}}_{5}^{+}$/CHO^+^) and 39 (K^+^/${{\rm{C}}}_{3}{{\rm{H}}}_{3}^{+}$), which are common fragments of most organic compounds and target surface contaminants (in the cases of Na^+^ and K^+^), are not considered diagnostic for specific species and are largely excluded from further interpretation.

During the E5 fly-by, the unique operational mode of CDA (Methods) resulted in lower-quality type II spectra with only a few spectra showing signal-to-noise ratios high enough to reliably detect organic functional groups, making quantitative analysis impossible. It is possible that organics of the same classes as those identified here were not observed in such high-noise spectra in the data, or that they were present in quantities below the detection limit.

### Aryl compounds

**Fig. 1:**
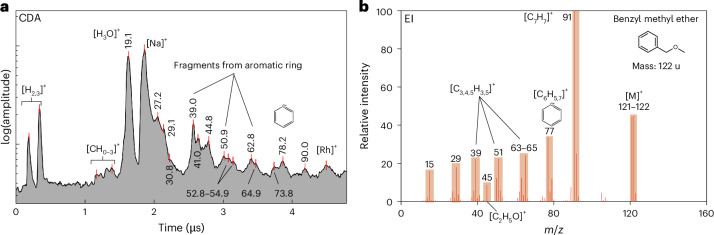
Archetypal CDA and EI mass spectra of an aromatic organic compound(s). **a**, CDA mass spectrum of a single plume ice grain characteristic of monocyclic aromatic compounds. **b**, An EI spectrum of benzyl methyl ether, which exhibits similar spectral features. Note that, for all figures, mass values are assigned to organic-related, and other notable, peaks to account for differences in the *x*-axis parameters between CDA and standard EI mass spectra.

As illustrated in Fig. 1a, the CDA spectrum exhibits spectral characteristics that are indicative of aryl group species. The peak at *m*/*z* ~ 77–79 represents either benzene or the phenyl cation [C_6_H_5–7_]^+^—as observed in E-ring ice grains^8,10^. The phenyl cations coincide with a peak at an *m*/*z* value of 90–91, indicative of the tropylium cation [C_7_H_7_]^+^. The spectral features at *m*/*z* values of 38–40, 49–52 and 62–65 are characteristic fragment species of single-ringed aromatics. Some of these species with variable C/H ratios correspond to [C_3_H_3_]^+^, [C_4_H_1–5_]^+^ and [C_5_H_1–5_]^+^ and are directly produced via fragmentation of the aryl ring^8,10,28,29^. The broad peak near an *m*/*z* value of 27, which extends to approximately to an *m*/*z* value of 31, may represent the oxygen-bearing species [CH_1–3_O]^+^, which is coincident with other O-bearing cations at *m*/*z* values of 43–45—[C_2_H_3,5_O]^+^. The peaks corresponding to specific fragment ions that are present in the EI mass spectrum of benzyl methyl ether (C_8_H_10_O), shown in Fig. 1b, correspond particularly well to the CDA spectrum.

### Aliphatic O-bearing compounds

**Fig. 2:**
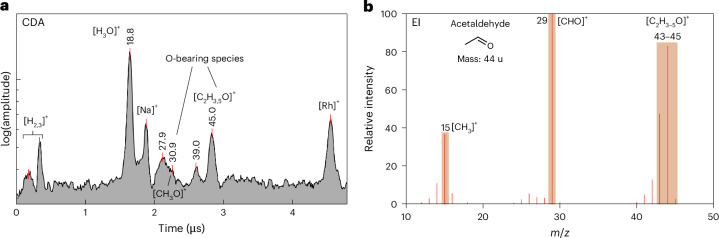
Archetypal CDA and EI mass spectra of an aliphatic O-bearing organic compound(s). **a**, CDA mass spectrum of a single ice grain indicative of an aliphatic O-bearing compound. **b**, The EI spectrum of acetaldehyde, which offers a good match to some spectral features observed here.

Figure 2a shows a CDA spectrum that corresponds to an aliphatic O-bearing species, probably a carbonyl group attached to a C_2_ organic (for example acetaldehyde or acetic acid). This spectrum exhibits a high degree of correlation with the EI spectrum of acetaldehyde (Fig. 2b). The O-bearing feature [C_2_H_4_O]^+^ at an *m*/*z* value of 44 in the EI spectrum is consistent with the feature at an *m*/*z* value of 45 [C_2_H_5_O]^+^ that is characteristic of O-bearing compounds in CDA spectra^10,27^. The absence of organic peaks at masses greater than an *m*/*z* value of 45 indicates that this may be a molecular peak.

**Extended Data Fig. 2:**
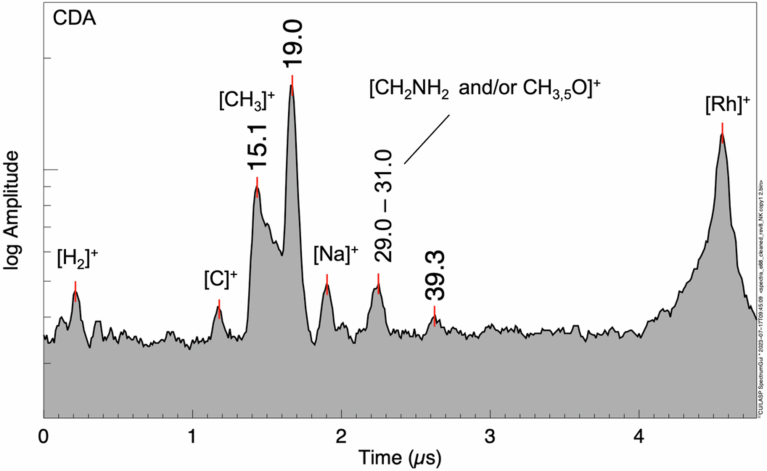
Single O-bearing CDA spectrum of Enceladus’s plume ice grain. Individual O-bearing spectrum of a plume ice grain exhibiting peaks at m/z 15 and 29-31. Both groups of mass spectra, the one with peaks m/z 15 and 29-31 and the other showing peaks at m/z 29-31 and 43-45, can be assigned to O-bearing compounds for example acetaldehyde or methanol, with differences in the fragmentation patterns attributed to different dynamics and structural heterogeneities between ice grains as described by Jaramillo-Botero et al.^57^. In this case, the differences with main text Fig. 1 can be explained by the characteristic ion of 45 u not surviving in this impact, and instead producing the coincident fragment cations of m/z 15 and 29-31.

The presence of a peak at *m*/*z* ~ 31, which corresponds to [CH_3_O]^+^, is a supporting feature of O-bearing species as discussed in ref. ^10^. Consequently, a few CDA spectra (Extended Data Fig. 2) that show a distinct feature at 30–31 without any peak at an *m*/*z* value of 45 are classified as O-bearing spectra.

### Esters (and/or alkene) compounds

**Fig. 3:**
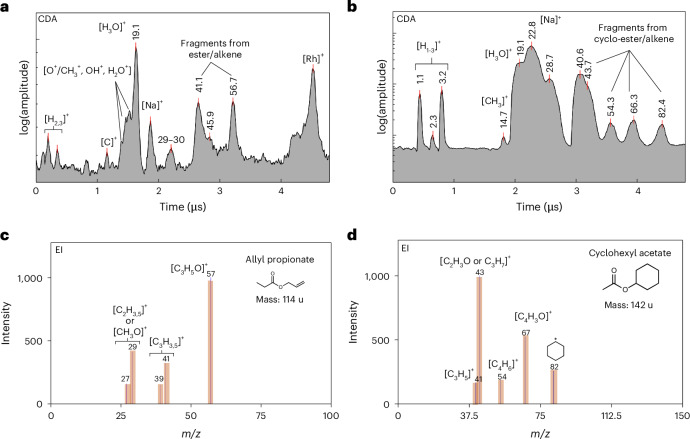
Archetypal CDA mass spectra of two individual plume ice grains containing an ester/alkene group and corresponding EI spectra. **a**,**b**, CDA mass spectra of potential aliphatic (**a**) and cyclic (**b**) ester species and/or alkene compounds in individual Enceladus plume ice grains. **c**,**d**, An EI spectrum of allyl propionate (**c**) and an EI spectrum of cyclohexyl acetate corresponding to aliphatic and cyclic ester/alkene species (**d**).

Figure 3a depicts a CDA spectrum obtained from a single ice grain that contained esters and/or alkenes. Features from organics at approximately *m*/*z* values of 41 and 57 are observed in CDA data owing to the high impact speed. These features have the potential to interfere with water-cluster species (Na^+^(H_2_O)_1,2_), which are commonly observed at lower impact velocities^8,10^. The EI spectrum shown in Fig. 3c of allyl propionate was found to be the best match to the observed CDA spectrum. The peaks at an *m*/*z* value of 40–41 and 56–57, which correspond to fragments from allyl propionate [C_3_H_3,5_]^+^ and [C_3_H_5_O]^+^ respectively, appear in both the CDA and EI spectra. The fragment cations thus provide constraints on organic structure, for which the most plausible classes are ester and/or alkene species.

Figure 3b,d shows a CDA and a corresponding EI spectrum of cyclohexyl acetate (C_8_H_14_O_2_; 142 u), a cyclic ester candidate that exhibits key CDA spectral characteristics. The peak at an *m*/*z* value of 82–83 is attributed to the fragment ion [C_6_H_10,11_]^+^, potentially produced by the cleavage of the bond between the ester and cyclic parts of the molecule. Two pairs of peaks at *m*/*z* values of 67 and 43 and *m*/*z* values of 54 and 41 potentially derive from the fragmentation of the molecular ion, relating to the acetate and cyclic parts of the molecule, respectively. Other candidate compounds are given in Extended Data Table 1).

### Ether (and/or ethyl) compounds

**Fig. 4:**
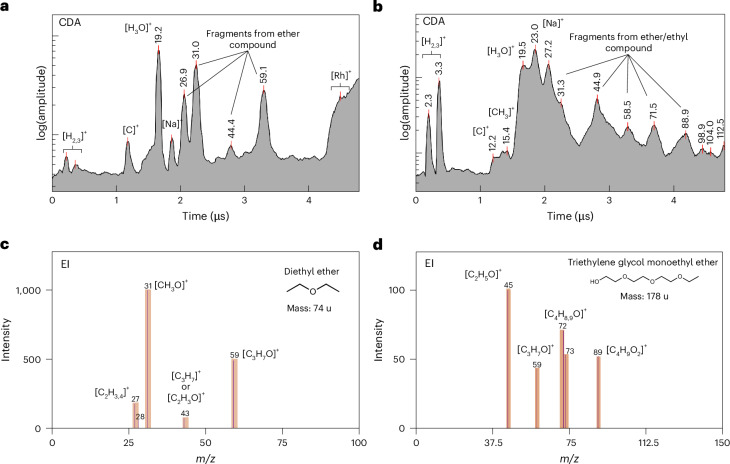
Archetypal CDA mass spectra of two individual plume ice grains containing an ether/ethyl group and corresponding EI spectra. **a**,**b**, CDA mass spectra corresponding to potentially short- (**a**) and long-chain (**b**) ether/ethyl compounds in individual Enceladus plume ice grains. **c**,**d**, EI spectra of diethyl ether (**c**) and triethylene glycol monoethyl ether (**d**), potential candidate compounds for the CDA spectra.

Figure 4a,b shows two CDA spectra of organic enriched plume ice grains, exhibiting a distinctive set of peaks at *m*/*z* values of 27, 31, 44–45 and 59. These features are well matched by the EI spectrum of diethyl ether (Fig. 4c) in addition to other potential candidate compounds (Extended Data Table 2). The two classes (Fig. 4) of organic compounds exhibit significant major peaks at *m*/*z* values of 31 and 59, corresponding to [CH_3_O]^+^ or [CH_3_NH_2_]^+^ and [C_3_H_7_O]^+^ or [C_2_H_5_(NH_2_)]^+^, respectively. In both instances, the ethyl group is a common moiety. The peaks at 43–45 are characteristic spectral features corresponding to each class of organic compound.

### N- and O-bearing moieties

**Fig. 5:**
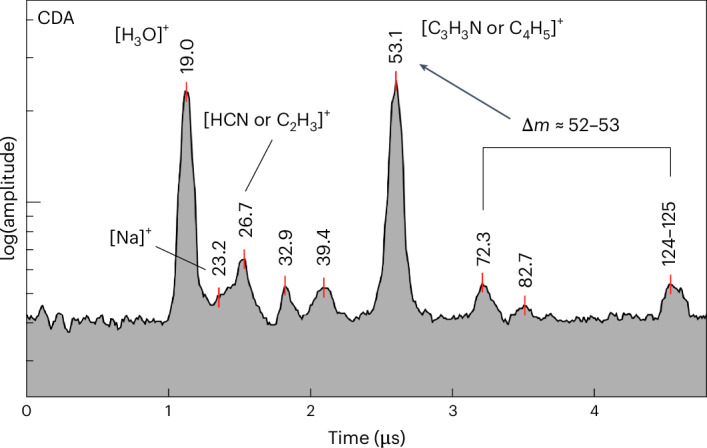
Archetypal CDA mass spectrum for N- and O-bearing species. CDA spectrum of a plume ice grain showing coincident cations that correspond to a species potentially containing nitrogen and oxygen. Examples of candidate species responsible for molecular ions include C_6_H_5_NO_2_, C_5,6_H_4,8_N_2_O_1,2_ and C_6,7_H_7,11_NO_2_. Their potential fragment species include C_4_H_6_N_2_, C_4_H_5_NO, C_4_H_9_N, C_3_H_5_NO, C_3_H_3_N, C_4_H_5_, C_3_H_3_, CH_4_O, H_2_N_2_, HCN, C_2_H_3_ and CHO.

**Extended Data Fig. 3:**
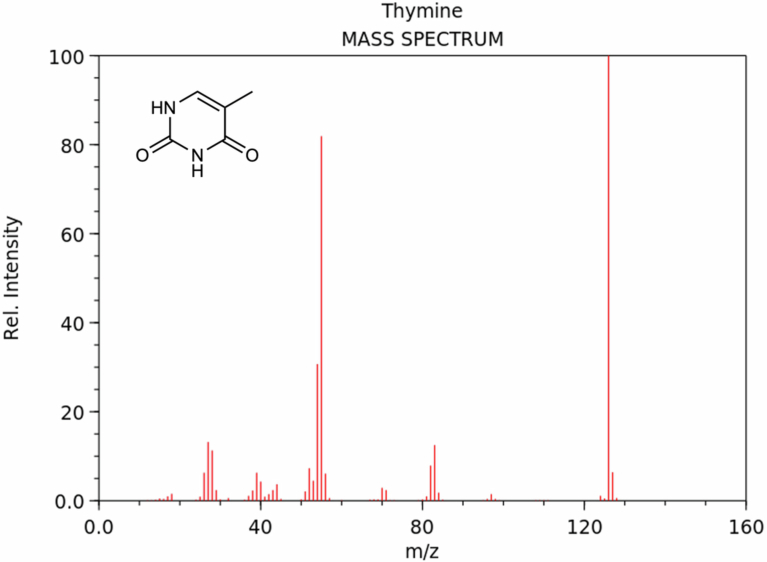
EI mass spectrum of Thymine compound from NIST relevant for N-, O-bearing species. Electron ionization mass spectrum of Thymine compounds from NIST online mass spectral library showing spectral features close to the features observed in CDA mass spectrum of N-, O-bearing species (Main Text Fig. 5).

Figure 5 shows a spectrum containing coincident cations at certain *m*/*z* values, which correspond to mass differences between other spectral features at higher masses. The base peak at an *m*/*z* value of 53 is attributed to the mass difference between peaks 124–125 u and 72 u. The cleavage of an N-heterocyclic ring can produce two separate fragments that contribute to the feature at an *m*/*z* value of 53: [C_4_H_5_]^+^ and [C_3_H_3_N]^+^. [C_3_HO]^+^ could also contribute to the base peak at 53 u, generated by other routes of fragmentation from larger molecules (Extended Data Table 3). The oxygen-bearing group potentially generates a fragment cation at an *m*/*z* value of 72–73 of [C_3_H_4,5_O_2_]^+^, which can further fragment into formyl [CHO]^+^ and its derivative cations at an *m*/*z* value of 31–33 [CH_3,5_O]^+^. The molecule containing both N- and O-bearing moieties could be cleaved to produce a spectral feature at an *m*/*z* value of 82–83 such as [C_4_H_4,5_NO]^+^ from the molecule. Species containing at least five carbon atoms (Fig. 5), and a variety of various N- and O-bearing moieties, including derivatives of pyridine, pyrimidine, maleic acid and nitriles (Extended Data Table 3), are potential candidates for these spectral features. No compounds with exactly matching EI spectra could be identified for these CDA spectra. However, some potential candidate molecules (for example, thymine, ethyl cyanoacrylate) with partially matching spectra are provided in Extended Data Fig. 3. Similar spectral features were previously observed in E-ring ice grain mass spectra obtained at lower velocities^10^.

## Discussion

This work examines the chemical composition of organic-enriched ice grains that were ejected into the Enceladus plume mere minutes before sampling by the CDA mass spectrometer during the fastest fly-by of Enceladus by Cassini (E5; ~17.7 km s^−1^). The high impact speed provides new insights into the composition of these freshly ejected grains because of the formation of cationic species that either did not form at lower impact speeds or were obstructed by water-cluster species that are not retained at these speeds^24^. We rule out any influence of post-impact chemistry on the interpretation of species detected in this work (Supplementary Figs. 1–3). In this study, we present the identification of aliphatic and cyclic ester/alkene and ether/ethyl moieties in freshly emitted Enceladean ice grains. In addition, some spectra show features indicative of mixed moieties, potentially N- and O-bearing moieties (Fig. 5 and Extended Data Table 3). The detection of aryl and O-bearing compounds in the plume ice grains is confirmed, as these were previously identified in the E ring at lower impact velocities^10^.

Recent modelling work on the possibilities for organic synthesis on Enceladus within hydrothermal systems is consistent with our detection of aromatics, esters, alkenes, aldehydes and low-mass N-bearing compounds^30^, with the exception of ether compounds, which recent work has not investigated. The presence of these compounds in fresh ice grains from Enceladus provides a number of implications for their formation and evolution in sea-floor hydrothermal systems, as well as for interactions with previously identified organic compounds in the synthesis of more complex organics.

### Aryls

**Fig. 6:**
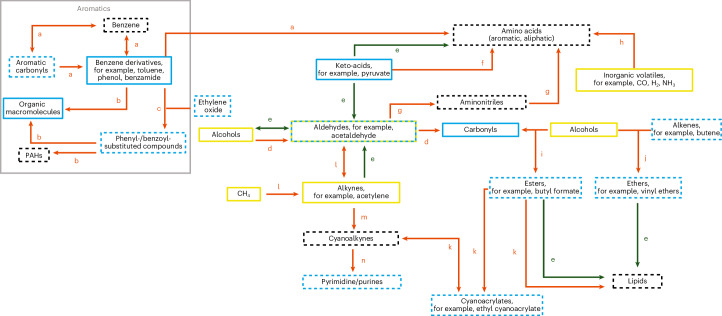
Potential chemical pathways between organic compounds on Enceladus. Potential chemical pathways between compounds detected in this and earlier works on Enceladus^4,5,8,10,17,65,66^, including both aqueous and ice-phase reactions, as well as those compounds that have not been detected to date, but would have astrobiological significance or provide pathways between other classes of compound. Legend: Blue boxes correspond to a CDA detection of the given functional group. Yellow boxes correspond to an INMS detection. Dashed boxes relate to those compounds potentially detected by CDA in this work, including both reliable detections and tentative suggestions. Solid boxes represent compounds that have potentially been detected in prior works, regardless of the confidence interval. Black boxes refer to compounds that have not yet been detected on Enceladus, but would be significant either in the context of astrobiology (for example amino acids) or as intermediates between other detected compounds (for example cyanoalkynes). The arrows between compound classes describe putative reaction pathways, with red arrows referring to abiotic pathways and green arrows representing biotic pathways. Note that some pathways would depend on ice–ocean exchange processes. The lower-case letters indicating pathways between compound classes denote the corresponding citation printed on the figure. a, refs. ^10,35^; b, refs. ^67–70^; c, ref. ^71^; d, oxidation; e, refs. ^72–74^; f, ref. ^75^; g, refs. ^76,77^; h, refs. ^78,79^; i, esterification; j, ref. ^80^; k, refs. ^81,82^; l, refs. ^70,83–85^; m, refs. ^48,85,86^; n, refs. ^49,87^.

It is currently unclear if the aryl compounds (aromatics) identified in this study are primordial—akin to those observed in carbonaceous chondrites—and have been leached from accretional material, or if they have been formed endogenously through hydrothermal reactions on Enceladus. It is impossible to determine isotopic ratios using CDA mass spectra, which could shed light on their origin, although future instruments may provide this capability. In the endogenous case, redox chemistry has the potential to facilitate the synthesis of aromatic compounds from a range of volatiles that have already been detected on Enceladus (Fig. 6). The possibility that aromatic-like compounds could be synthesized in freeze–thaw cycles at the water–ice interface is unlikely due to the limited formation potential of carbon radicals, which are important for the formation of polycyclic aromatic hydrocarbons (PAHs) in terrestrial sea ice^31–33^, and the fact that significant fractions of aromatic organics are excluded from the ice phase during freezing^34^.

The presence of an aryl group linked with alkyl or O-bearing moieties may offer avenues for the synthesis of biologically pertinent organic compounds^10,35^. Aryls with certain moieties are reactive under hydrothermal conditions, facilitating their transformation into more stable species such as benzene and phenol^36,37^. Single-ring aromatics play a pivotal role in the organic chemistry of hydrothermal systems on Earth’s sea floor, serving as primary sources of further organic compounds^38–41^. The presence of aryl groups in the fresh plume suggests that these compounds retain their aromatic structure in hydrothermal sites before transport through the ocean and incorporation into ice grains at the water surface. The detection of organics directly in the plume rules out space weathering as the sole production pathway, and the study in ref. ^30^ shows that cold aqueous chemistry is also ruled out; therefore, the grains are fresh, unaltered and proof of survival through ocean transit and plume emission of compounds, indicative of warm hydrothermal chemistry. We discount an aliphatic origin of these mass spectra, as aromatic molecules generally create high yields of low-mass ions only in high-speed impacts, whereas aliphatics experience extensive fragmentation^42^.

### O-bearing organics

Among the aliphatic O-bearing moieties (Fig. 2), we posit that aldehydes represent a moiety present in freshly ejected ice grains from Enceladus. Aldehydes may have been accreted in the building materials of Enceladus since they are relatively abundant in comets^43,44^. Aldehydes represent intermediates in the redox pathway from simple hydrocarbons to carboxylic and amino acids^45^. The presence of acetaldehyde on Enceladus would offer possibilities for synthetic routes towards more complex organics essential for life^46,47^. Acetaldehyde is also linked to acetylene—detected in the plume by INMS^4,17^—in chemical cycles within prebiotic hydrothermal systems^48,49^. Ethylene oxide (C_2_H_4_O) could also be a potential candidate for this type of spectral feature, which could catalyse the formation of polymers with multiple end groups (for example -CH_2_, -OH). For example, ethylene oxide can assist alkylation of simple aryl compounds (for example benzene) through Friedel–Crafts-like reactions, resulting in the formation of aryl-substituted alcohols under hydrothermal conditions^30^. This process could subsequently lead to the synthesis of more complex organics such as PAHs or the macromolecular species detected in Enceladean ice grains^8^.

### Ester/alkene and ether/ethyl

This detection of aliphatic and/or cyclic ether, ester and alkene moieties in plume ice grains complements previous identifications of these molecules in other planetary bodies across the solar system. Ethers and esters are rarely found in comets^50^, but they occur as bridges between aromatic moieties of insoluble organic matter in carbonaceous chondrites^51^. This suggests that ether and ester moieties can be formed via aqueous/hydrothermal reactions in carbonaceous chondritic bodies. The presence of these compounds, and the possibility for their synthesis in the hydrothermal systems of Enceladus is relevant for planetary habitability, given their role in terrestrial biological contexts, as shown in Fig. 6. The isotopic analysis of diether lipids from the Lost City Hydrothermal Field in the mid-Atlantic Ocean provides insight into the abiotic or biotic origins of carbon in hydrothermal systems^52^, emphasizing the significance of such analysis for future missions targeting ocean world sampling. It has been demonstrated that ester compounds can form under reductive hydrothermal conditions from lipid precursors in the presence of ammonium ions, a realistic scenario for the water–rock interface of Enceladus^53^. Such esters are stable under hydrothermal conditions and retain a distinct abiotic signature, namely no even carbon number predominance. The detection of both phosphates^9^ and esters on Enceladus is of significance for astrobiology, offering potential pathways towards important biomolecules^54^. Alkenes are intermediates in a variety of reactions between more abundant classes of organics in submarine hydrothermal systems^55^. Such compounds are involved in hydration, oxidation, and dimerization reactions under these conditions. The presence of alkenes on Enceladus probably diversifies the chemical reactions accessible at hydrothermal sites.

### Mixed N- and O-bearing groups

For compounds with multiple moieties tentatively identified in this work (Extended Data Table 3), possible fragmentation pathways are presented in Fig. 5. Candidate compounds include derivatives of pyrimidine, pyridine, acetonitrile and maleic acid, among others. It is noteworthy that acetonitrile and various amine derivatives have a particular affinity for synthesis under Enceladean conditions^30^. The cyanate ion was also detected in INMS data in ref. ^17^ and could react with acetylene, providing pathways towards larger, more complex N-bearing species. It is also possible that the cyanate species detected by INMS were produced by the fragmentation of acetonitrile, a molecule that could be related to the candidate species for this type of spectrum.

## Conclusions

We report the presence of organic moieties, including the detection of esters/alkenes, ethers/ethyl and N- and O-bearing species by Cassini’s CDA, in ice grains freshly ejected from Enceladus and sampled at the highest fly-by speed (17.7 km s^−1^). These new organic functional groups enable further avenues for hydrothermal chemistry, in addition to those pathways previously postulated^5,8,10,13,14^ (Fig. 6). We also confirm the presence of previously identified^10^ aromatic and O-bearing species, and impose new constraints on their origin. This work demonstrates that such moieties in these freshly ejected ice grains are probably derived from within Enceladus rather than from space weathering^22^ during their lifetime in the E ring. Although the possibility of post-impact plasma chemistry cannot be ruled out, the species identified in this work are solely independent of such processes at these velocities (Supplementary Information section 1). The data obtained by INMS during the E5, E17, E18 and E21 fly-bys^5,17^ are in good agreement with the results reported here (Extended Data Table 4). The detected moieties in the plume further hint at an organic-enriched subsurface, with a diverse range of reaction pathways expanding both the known and potential chemical space of the Enceladus ocean.

## Methods

### E5 fly-by and data collection

Cassini’s CDA recorded 1,519 distinct TOF mass spectra of ice grains in the close vicinity of Enceladus over approximately 6 minutes during its E5 fly-by. The fly-by was performed at 17.7 km s^−1^ and the closest approach occurred at 2008-283T19:06:40 UTC (Coordinated Universal Time) at a distance of 21 km from the fringe of the tiger stripes near Alexandria. The fly-by saw Cassini traverse the part of the plume with the highest number density of ice grains. The mass spectra obtained during the E5 fly-by of Enceladus were recorded in a distinctive operational mode of CDA. This fly-by was the only close approach to Enceladus during which a specially modified flight software (FSW 12.0) was employed by the instrument, enabling a spectrum recording rate of up to five per second, rather than the rate of one per second in the nominal configuration. Under these conditions, however, the recorded mass range spanned 2–110 u (or, in a few cases, up to 125 u), a reduction from the standard 1–200 u. In addition, the sampling rate was also reduced (one data point every 20 ns instead of every 10 ns), further limiting the mass resolution^7^.

In most cases, the spectrum recording of the incident water ice grain was triggered by the detection of high-amplitude mass lines corresponding to the short flight times of fast hydrogen ions H^+^, ${{\rm{H}}}_{2}^{+}$ or ${{\rm{H}}}_{3}^{+}$. In addition, there are some instances when the spectra recording started upon the impact of the ice grain at the target even before the first H^+^ ions could have arrived at the CDA’s multiplier, potentially due to a higher particle impact rate that increased the noise level, which prematurely triggered the instrument. The average value of the stretch parameter (for detail, see ref. ^7^ and Supplementary Fig. 4) of the instrument is approximately 506 ns. This mechanism of spectrum recording is described in detail in previous works^6–8,10,23^. The mass range of the recorded spectra generally extends to an upper limit of 103 u, with some exceptions where it reaches 125 u. The water-cluster features [H_3_O]^+^(H_2_O)_1,2,3…_ that appear in lower-speed spectra^8,10^ are not observed due to the extremely high impact speeds encountered during the E5 fly-by relative to E ring traversals of the Cassini spacecraft. In all CDA spectra shown in this work, the impact of ice grains at such velocities generates rhodium ions [Rh]^+^ from the Chemical Analyser target material.

### Classification of type II spectra

The CDA spectra shown in this work exhibit a peak at an *m*/*z* value of 19, corresponding to the hydronium cation [H_3_O]^+^ without any additional water-cluster features, as are typically detected in E-ring ice grain spectra recorded at much lower velocities. The study of ref. ^7^ previously classified these spectra into three categories, corresponding to different populations of ice grains originating from Enceladus. (1) Type I: almost pure water ice with only trace amounts of organics. The hydronium peak [H_3_O^+^] at an *m*/*z* value of 19 dominates these spectra, with no pronounced peaks between 28 and 29 u. (2) Type II: organic-enriched ice grains, dominated by the hydronium peak [H_3_O^+^], but also containing pronounced features between 28 and 29 u. (3) Type III: salt-rich grains. Type III spectra are dominated by Na^+^ and NaOH and/or NaCl and/or ${{\rm{Na}}}_{2}{{\rm{CO}}}_{3}/{\rm{NaHCO}_{3}}$ cluster peaks, exhibiting neither a significant hydronium feature nor peaks at 28–29 u.

In this work, we focus only on type II mass spectra, with a slight modification in the criterion set in ref. ^7^. In our reinvestigation, we include spectra with peaks between an *m*/*z* value of 25–33 and/or an *m*/*z* value of 39–46, corresponding to hydrocarbons and oxygen- and nitrogen-bearing species. In total, 409 spectra are classified as type II, and these are selected for in-depth analysis. Five compositional subgroups of organic-enriched type II grains (86 spectra) are identified with a unique set of peaks in their spectra (Supplementary Table 1). A list of possible fragment ions corresponding to the mass lines observed in these spectra is given in Supplementary Table 2.

In this work, we have analysed the full dataset of the E5 fly-by in detail. The type II spectra recorded during this particular fly-by are generally of lower quality due to the higher spectral recording rate (5 s^−1^), lower mass range (125 u) and reduced sampling rate (one data point every 20 ns instead of every 10 ns) in this unique operational mode of CDA. We find only a small number of spectra with signal-to-noise ratios large enough to identify features characteristic of certain organic functional groups. The data quality was not only generally poor, but also varied across the plume traversal. It is possible that organics of the same classes as those identified here were not observed in such high-noise spectra, or that they were present in quantities below the detection limit. Quantitative analysis is not possible under these non-optimal conditions; therefore, this paper aims solely to identify organic compounds wherever possible.

One interesting aspect that should be mentioned is the case of aromatic species. Aromatics seem to be enhanced in type II ice grains relative to the other organic subtypes detected at 18 km s^−1^. This could be because they are more stable across all impact speeds, or form more abundant characteristic fragmentation products than other organics. As described previously, however, we cannot reliably draw conclusions about the relative proportions of aromatics in the larger dataset.

### Electron ionization mass spectra

In this work, EI mass spectra (Extended Data Tables 1–3), extracted from Massbank Europe, NIST and MoNA, have been used to aid spectral interpretation following the methodology of ref. ^27^. High-velocity (that is, 18 km s^−1^) impacts provide such energy that water-cluster formation is inhibited in the plasma cloud post impact. With the exception of the hydronium [H_3_O]^+^ ion at an *m*/*z* value of 19, the standard patterns of water clustering^24^ are not observed in these CDA spectra. As EI spectra are not recorded in the presence of a matrix, unlike the analogue experiment LILBID, they offer a strong match to CDA spectra in these high-velocity cases. Even at the highest laser power densities, water clusters still appear in LILBID and complicate the identification of organic features in the spectra.

Both EI and impact ionization are considered ‘hard’ ionization methods, in which a large number of fragment ions are formed, reducing the intensity of the molecular ion peak, particularly in the case of higher impact velocities^56^. We note that the study in ref. ^56^ observed a number of quantitative discrepancies between EI and impact ionization mass spectra obtained from dust accelerator experiments with polypyrrole-coated anthracene. This work, however, used dust particles with little to no water content, whereas CDA detects ice grains dominated by water. Similarly, their experiments investigate only PAHs with fused aromatic rings; here we consider single-ringed aromatic compounds which, even if part of a larger molecular structure, have a number of substituted groups on the ring. Furthermore, our findings illustrate that aryl and O-bearing moieties remain stable even at the highest impact speeds of ~18 km s^−1^, probably due to the protective nature of the ice matrix. The abundance of characteristic low-mass fragment ions from polypyrrole-coated anthracene has been shown to rapidly decline above 15 km s^−1^ in experiments with dust accelerators^56^, indicating that the ice matrix plays a pivotal role in retaining spectral characteristics associated with aromatics. While fragmentation is influenced by the size and structure of the ice matrix at low velocities, such a dependence is insignificant at high impact speeds. The shielding role of the ice matrix is largely uniform above a certain threshold velocity, which is unique to the embedded organic molecule^57–59^.

Species with high ionization energies appear in both EI and impact ionization mass spectra, owing to the excess energy available in each ionization method^60,61^. In both cases, fragment ions convey crucial structural information in the mass spectra. In EI, minor peaks adjacent to *m*/*z* values of major fragment ions can also be observed, which assists in the identification of broad peaks in CDA mass spectra that occur due to its higher noise level than laboratory mass spectrometers. CDA and INMS often detect the same organic species, as INMS can also be triggered by the impact of incident ice grains, suggesting that similar fragmentation pathways are accessible by both methods^8^. INMS is sensitive to neutral fragments, offering a useful mode of comparison.

Note that LILBID is a crucial method for interpretating mass spectra of ice grains emitted by icy ocean worlds in the outer solar system at lower impact velocities such as those expected for Europa Clipper and its SUrface Dust Analyser^62^. See Supplementary Fig. 3 for further examples of the behaviour of water clustering in both CDA and LILBID mass spectra. The high impact velocities experienced in the E5 fly-by can guide the interpretation of mass spectra from other fast fly-by and interplanetary missions such as the Demonstration and Experiment of Space Technology for INterplanetary voYage with Phaethon fLyby and dUst Science (DESTINY+) by the Japan Aerospace Exploration Agency (JAXA), which will also carry an impact ionization mass spectrometer—the DESTINY+ Dust Analyser^63^—for the compositional analysis of interplanetary and interstellar dust particles.

### Assignment of species to CDA spectra

The highest-impact-speed CDA spectra (Figs. 1–5) of freshly ejected ice grains provide new constraints on the structure of different embedded species. We constrain the structure of aryl species by the presence or absence of a peak at an *m*/*z* value of 91, which would correspond to tropylium cation (Fig. 1a). The presence of this peak indicates a more benzyl-like nature of aromatics in ice grains, whereas its absence implies phenyl-type species^8,10^. Certain O-bearing species can produce the methenium cation at an *m*/*z* value of 15, coincident with a formyl cation at an *m*/*z* value of 29–31. It is clear that the methyl groups must exist in some form and survive impact at such high velocities. The combined spectral features of Extended Data Fig. 2, with peaks at *m*/*z* values of 15, 29–31 and 45, are likely to correspond to [CH_3_]^+^, [CH_1-3_O]^+^ and [C_2_H_5_O]^+^. The best EI spectral match to the observed cationic distribution is the acetaldehyde (C_2_H_4_O) spectrum, which also correlates strongly to earlier predictions of the Enceladus chemical inventory^4,10^.

In addition to the characteristic identifiers of aromatic and O-bearing species observed in these mass spectra of plume ice grains, we also detect characteristic spectral features of esters, alkenes, ethers, ethyl and N- and O-bearing compounds. The CDA mass spectral features of esters/alkenes and ethers/ethyl groups are compared with EI spectra using online open-source mass spectral databases. Two different sets of peaks correspond to ester/alkenes (Fig. 3 and Extended Data Table 1). (1) In this case compounds are included where the peaks at *m*/*z* values of 41 and 56 should have an intensity within 30% of each other in EI spectra. In addition, if present, the intensity of the 43-u feature should be 20% less than the peak at 41 u. (2) In this case, compounds are included whose EI spectra show a set of peaks at *m*/*z* values of 41–43, 54, 67 and 82. The relative intensities of these peaks should lie within 20% of each other. In addition, no other peaks should appear in their spectra with a relative intensity greater than 20%. This logic is shown graphically in Extended Data Fig. 1.

In the ether/ethyl group, candidate compounds are included if their EI spectra show two different sets of peaks (Fig. 4 and Extended Data Table 2). (1) in this case, peaks at *m*/*z* values of 31 and 59 are present with a relative intensity greater than 30%. In addition, a peak at an *m*/*z* value of 43–45 can be present only if any peak at an *m*/*z* value of 41 u occurs with less than half its intensity. (2) In the second case, compounds are included which show peaks at *m*/*z* values of 43–45, 58–59, 71–72 and 88–89 in their EI mass spectra. In both groups (esters/alkene and ether/ethyl), a few additional candidates are given related to other, typically more exotic and unlikely, moieties, for example halides. There is another group of CDA spectra corresponding to N- and O-bearing compounds, which show a set of peaks at *m*/*z* values of 124–125, 82–83, 71–72, 31–33 and 26–27, in addition to a significant peak at an *m*/*z* value of 52–54 (Fig. 5). For these mass spectral features, no representative EI mass spectra could be found in freely available online databases. However, the observed fragment ions can be traced back to possible parent compounds^28^, shown in Fig. 5 and Extended Data Table 3.

### Sampling and composition of ice grains

The spectral features observed in this work are attributed to compounds with masses below ~125 u, but we cannot completely rule out that the ion species analysed here are moieties of much larger molecules^8^ that are fragmented during high-speed impacts. This could mean that some fragmentation pathways of large molecules may require energies accessible only at higher impact speeds, leading to new observed fragment ions in this work. Alternatively, some species (ether/alkene, ester/ethyl and N- and O-bearing) detected in this work may become unstable due to space weathering effects, explaining their non-detection in E-ring mass spectra in ref. ^8^. The lack of detection in the E ring can be due to one of, or a combination of, the following factors:

1. Spectral features in E-ring spectra were obscured by water-cluster species that do not form at the high impact velocities we consider in this work.
2. Space weathering effects lead to the dissociation of organic compounds and the loss of volatile components in E-ring ice grains.
3. Some fragment ions identified in this work can be produced only at the high impact velocities encountered in the E5 fly-by.

In this distinct operational mode of CDA, the mass range of the instrument does not extend to masses >120 u, where indicators of macromolecular species were previously observed. For these reasons, we report the detection only of specific functional groups, and suggest low- or intermediate-mass candidate compounds (mostly species of less than 150 u) that provide useful constraints on molecular structure.

In these datasets, we can infer structural constraints on the organic compounds responsible for the CDA mass spectra, but the complexity of high-speed impacts means that the absolute identification of molecules is challenging. In such high-speed (>17.7 km s^−1^) collisions associated with impact ionization, the distribution of energy across the organic species within the impact cloud is non-uniform, compared with the better-characterized EI technique—meaning that, while fragments produced are similar between the two ionization methods, they do not necessarily occur in the same abundances. Similarly, due to limitations of the CDA software, which was not originally designed for the high fluxes of ice grains encountered during plume fly-bys, the high impact velocities, the quality of the mass spectra and the discrepancies between peak amplitudes in the CDA spectra and example EI spectra, concentrations of candidate compounds cannot be inferred in the bulk ocean. The CDA spectra detailed in each figure (Figs. 1–5) were generated by the impact of a single ice grain, rather than averaged and co-added, as were often used in previous works^6,8,9,13^. The recent work in ref. ^64^ demonstrated that the compositional analysis of single ice grains is an important mode of sampling at ocean worlds. Not only does it yield relatively high-quality spectra, with distinct peaks generally more observable than in co-added spectra, but it also acknowledges the inhomogeneity of ice grains, where bulk ocean constituent compounds can vary significantly in concentration between different ice grains. Co-added spectra are not conducive to the detection of compounds that are generally present in low quantities in the bulk ocean, but incorporated into some ice grains with elevated concentrations. Such scenarios would not be detected by analysis of co-added spectra alone. Each individual ice grain thus provides a unique window into the potential habitability of the Enceladus subsurface.

## Supplementary information

Supplementary Information Supplementary Discussion, Figs. 1–4 and Tables 1 and 2.

Supplementary Data 1 Raw data for LILBID spectrum of allyl propionate analogue to the CDA higher impact speed spectrum.

Supplementary Data 2 Raw data for LILBID spectrum of allyl propionate analogue to the CDA lower impact speed spectrum.

## Figures and Tables

**Extended Data Table 1:**
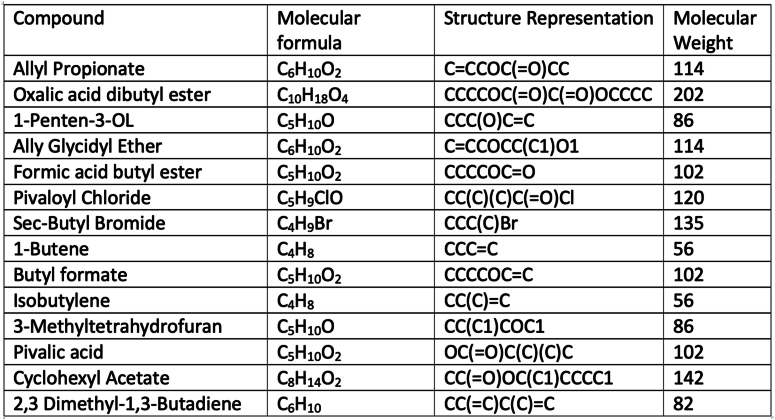
Ester/alkene group compounds

Potential ester/alkene group compounds responsible for the mass spectra shown in main text Fig. 3. Structural representation is based on molecular-input line-entry system (SMILES)^88^.

**Extended Data Table 2:**
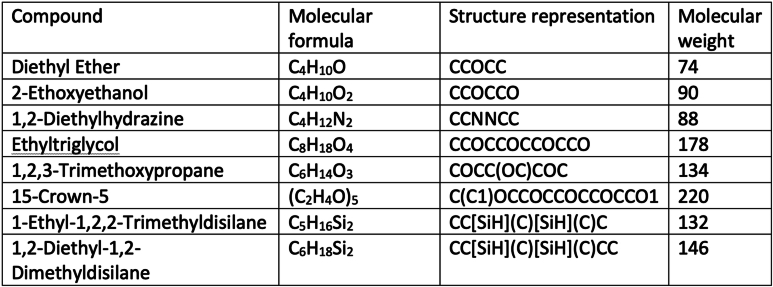
Ether/ethyl group compounds

Potential ether/ethyl group compounds responsible for the mass spectra shown in main text Fig. 4. Structural representation is based on molecular-input line-entry system (SMILES)^88^.

**Extended Data Table 3:**
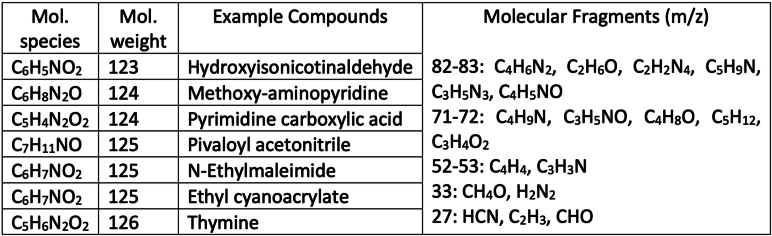
Tentative candidate species showing spectral features corresponding to N-O-bearing species

Tentative candidate species corresponding to the spectral features indicative of N-O-bearing species mentioned in Fig. 5. No representative spectra were found in freely-available online databases for these compounds. However, by tracking fragmentation patterns, the most likely parent compounds can be inferred based on the work of McLafferty and Turecek^28^.

**Extended Data Table 4:**
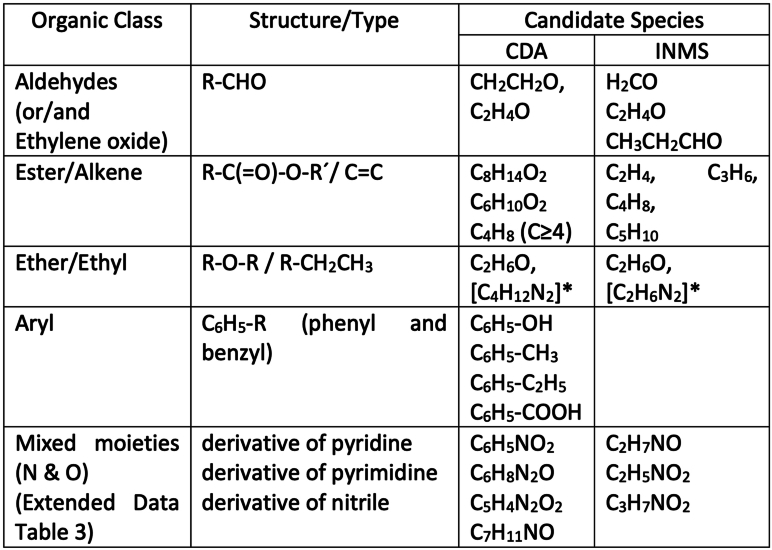
Classes of organic compounds detected by CDA in freshly ejected ice grains

Classes of organic compounds detected by CDA in freshly ejected ice grains during Cassini’s fastest flyby of Enceladus. A comparison of example species detected by CDA and INMS is also given^4,5,8,10,17,65,66^. *N-bearing compounds as potential candidates for the ethyl-like spectra are given in this category.

## Data Availability

All CDA data used for this work are listed in Supplementary Table 1 and are archived on the Planetary Data System Small Bodies Node (PDS–SBN), at https://sbn.psi.edu/pds/resource/cocda.html. EI data were obtained from NIST (https://chemdata.nist.gov/), MassBank Europe (https://massbank.eu/MassBank/), PubChem (https://pubchem.ncbi.nlm.nih.gov/) and MoNA (https://mona.fiehnlab.ucdavis.edu/) freely available online databases. The source data used to compile Supplementary Fig. 1a,b are provided as Supplementary Datasets 1 and 2, respectively.
