# Supplementary material for: Detection of organic compounds in freshly ejected ice grains from Enceladus’s ocean
Source: Nat Astron. 2025 Oct 1;9(11):1662–71. doi: 10.1038/s41550-025-02655-y (PMC12626887; doi:10.1038/s41550-025-02655-y)
Supplement: Supplementary file 1 — Supplementary Discussion, Figs. 1–4 and Tables 1 and 2. [file 41550_2025_2655_MOESM1_ESM.pdf]

nature astronomy

Supplementary information

https://doi.org/10.1038/s41550-025-02655-y

Detection of organic compounds in freshly ejected ice grains from Enceladus's ocean

In the format provided by the authors and unedited

Supplementary Information
Detection of Organic Compounds in Freshly Ejected Ice Grains from Enceladus's Ocean

1. On the possibility of post-impact chemistry

The high-speed mass spectra obtained by CDA during the E5 Cassini flyby of Enceladus are distinct from spectra obtained at lower speeds in Saturn's E ring. At high impact velocities (> 12 km/s), CDA spectra could show water dissociation products (e.g., H^+, O^+). However, the extent of clustering (particularly water clustering – i.e. the formation of ions of the form $[H_3O]^+(H_2O)_{n=1,2,3\ldots}$) is significantly reduced. Such hypervelocity impacts create an extremely destructive plasma environment where the fragmentation of compounds dominates over the formation of clusters. Lee et al.[89] and Nouzák et al.[90] observed that species in the impact plasma cloud move away from the impact location very fast with a mean expansion speed of about 21 km/s. In this regime, the plasma cloud becomes a repulsive region (particularly at an impact speed ≈ 18 km/s) for species where ions drive away from each other rapidly, which limits the potential for synthesis of new species as a byproduct of plasma/oxygen chemistry. It remains a possibility that some degree of recombination of fragment species in the plasma cloud could take place at high impact velocities before the rapid expansion phase, but we do not expect this to have a major influence on the identification of organic species in this and earlier works with CDA spectra.

We further used our LILBID facility and recorded allyl propionate spectra to simulate analogue CDA spectra with characteristic spectral signatures of ester/alkene compounds at higher (> 12 km/s) and lower (< 10 km/s) impact velocities. From these measurements, we successfully demonstrate that the identification of characteristic spectral features of the detected compounds in this work is independent of water-related species. LILBID spectra of allyl propionate at higher (left panel) and lower (right panel) velocities show mass lines at m/z 41 ($C_3H_5^+$) and 57 ($C_3H_5O^+$), which are used as diagnostic spectral features in CDA spectra of this type of compound. Both spectra show that these identifiers are independent of impact speed and hence independent of any oxygen/reactive chemistry. Therefore, the organic-related spectral features identified in this work are derived from species in plume ice grains and they are not the product of reactivity in the plasma cloud post-impact.

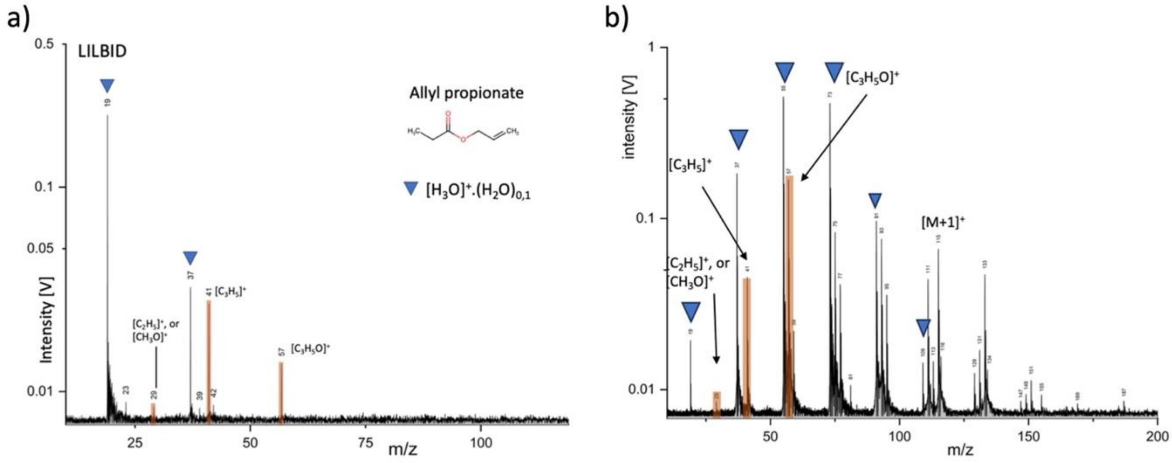

Supplementary Figure 1. LILBID spectra of allyl propionate, a candidate compound for the spectral features observed in this work. The panel (a) spectrum is recorded at a simulated velocity of >12 km/s, whilst the panel (b) spectrum is recorded at a simulated velocity of < 10 km/s. Key spectral features of allyl propionate are highlighted in orange.

As reported in Postberg et al.[32] and Magee & Waite[31] INMS observed a variety of fragmentation products from ice grain impacts during the E5 flyby (≈ 18 km/s) but, beyond reactions with the titanium wall of the chamber, no significant recombination products were observed here. Whilst the possibility of such plasma chemistry cannot be completely ruled out, it's limited role in previous analyses of mass spectral data from Cassini argues for a similar approach here. In Supplementary Figure 2, we show spectra of the aromatic (left) and O-bearing (right) compounds detected at lower velocities[1] (upper panels) and at higher impact velocities (E5 data, this work; lower panels). The spectra show the same characteristic spectral features of aromatic and O-bearing compounds at the higher impact speeds (≈ 18 km/s; lower panels) as also observed at lower impact speeds[10] (top panels), demonstrating the lack of oxygen/reactive chemistry at higher impact velocities (≈ 18 km/s). The characteristic fragment cations (identifiers) for aromatic and O-bearing compounds at higher and lower impact speeds are similar despite the differences in peak intensities and water clustering owing to the distinct impact speeds. These results demonstrate that between these speed regimes (6-10 km/s and 18 km/s), there are no significant differences in plasma chemistry. Therefore, this has no significant influence on the identification of similar compounds at different velocities.

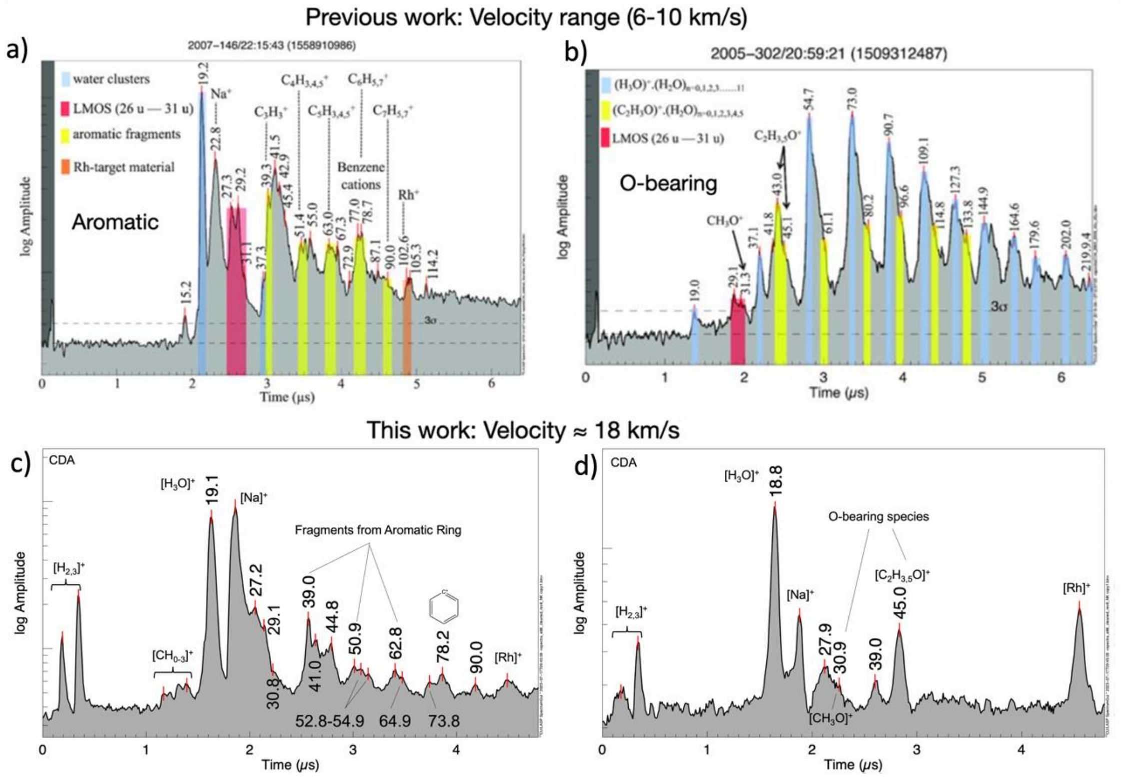

Supplementary Figure 2. CDA mass spectra exhibiting characteristic fragments of aromatic compounds (a) and O-bearing (b) compounds obtained from Saturn's E ring at lower impact speed[10] and higher impact speed (this work; panels c, d). Reproduced from Khawaja et al.[10]

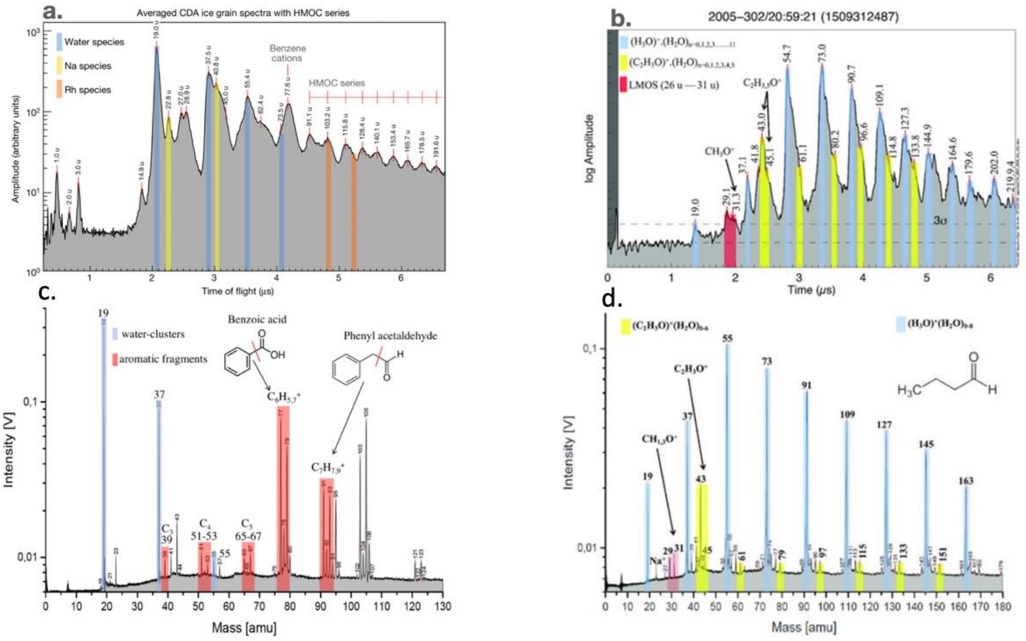

Supplementary Figure 3. CDA mass spectra published in refs. 8, 10. These spectra indicate the presence of aromatic and O-bearing fragments in E ring ice grains, and also provide a useful demonstration of water clustering behaviour at low velocities both in CDA and laboratory-simulated analogue LILBID spectra.

2. CDA Instrument Parameters

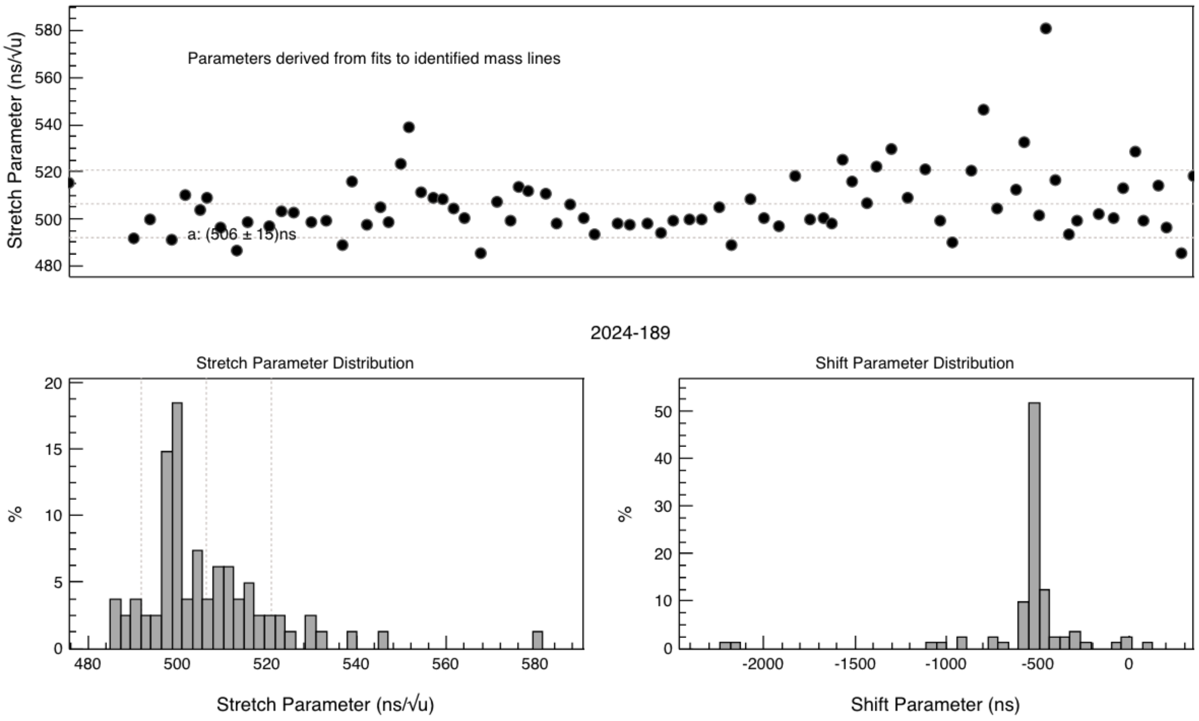

Supplementary Figure 4. Instrument performance that shows the distribution of the stretch (average value ≈ 506 ns) and the delay/shift parameter (average value ≈ 500 ns). See Postberg et al.[7] for more detailed discussion of instrument performance.

3. Spectra Statistics, Fragment Ions, and Event IDs

Classification	Series of characteristic spectral features (m/z)	Number of spectra
Aryl	48-53, 61-65, 76-79	51
Oxygen-bearing	(either) 29-33, 43-47<br>(or) 14-15, 29-33	18
Ester/alkene	(either) 41, 56-57<br>(or) 43-45, 53-55, 66-67, 81-83	6
Ether/ethyl	(either) 27-29, 31, 43-45, 59<br>(or) 43-45, 58-59, 71-72, 88-89	7
N+O-bearing	27, 31-33, 52-53, 71-72, 82-83	4

Supplementary Table 1. Number statistics of Type II spectra in each subgroup. Based on archetypal spectra in each subgroup, a series of characteristic spectral features (m/z) is inferred in each case.

Functional group	m/z	Possible fragment ions	CDA Spectra Event IDs
Aryl	12-15	$[CH_{0-3}]^+$	1421, 1562, 1569, 1629, 1729, 1785, 1809, 1853, 1997, 2016, 2049, 2065, 2110, 2279, 2665, 2808, 2989, 3056, 3213, 3326, 3353, 3395, 3578, 3720, 3785, 3809, 3838, 4156, 4270, 4483, 4538, 4667, 4773, 4809, 4864, 5109, 5406, 5827, 5966, 6008, 6023, 6065, 6545, 6532, 6564, 7099, 15348, 16079, 16698, 16793, 17496
	27-31	$[C_2H_{3-5}]^+$	
	38-40	$[C_3H_{2,3,4}]^+$	
	48-53	$[C_4H_{1-5}]^+$	
	61-65	$[C_5H_{1-5}]^+$	
	76-79	$[C_6H_{5,6}]^+$	
	91	$[C_7H_7]^+$	
O-bearing	14-15	$[CH_3]^+$	1825, 1905, 2991, 3309, 3512, 3530, 3804, 3855, 4154, 4138, 5141, 5142, 5267, 5522, 6305, 13536, 15868, 16192
	29-33	$[CH_{1-3}O]^+$	
	43-47	$[C_2H_{3,5}O]^+$	
Ester/alkene	15-18	$[CH_3]^+$, $[O]^+$, $[OH]^+$, $[H_2O]^+$	1978, 2211, 3454, 3643, 4756, 17791
	27-30	$[CH_3O]^+$, $[C_2H_{3,5}]^+$	
	39-41	$[C_3H_{3,5}]^+$	
	43-45	$[C_2H_{3-5}O]^+$	
	53-55	Cyclic-derived	
	56-57	$[C_3H_5O]^+$	
	66-67	Acetate-derived	
	81-83	$[C_6H_{9-11}]^+$	
Ether/ethyl	27-29	$[CH_3O]^+$, $[C_2H_{3,4}]^+$	1617, 2826, 3091, 6071, 6109, 14697, 15258
	31	$[CH_5O]^+$, $[CH_3NH_2]^+$	
	43-45	$[C_2H_{3-5}O]^+$	
	58-59	$[C_3H_7O]^+$, $[C_2H_5(NH_2)]^+$	
	71-72	Ether-derived	
	88-89	Ether-derived	
N+O-bearing	27	$[HCN]^+$, $[C_2H_3]^+$	4395, 15078, 16815, 17447
	31-33	$[CH_5O]^+$, $[CH_3NH_2]^+$, $[CH_4O]^+$	
	39	$[C_3H_3]^+$, $[K]^+$	
	52-53	$[C_3H_3N]^+$, $[C_4H_5]^+$	
	71-72	$[C_4H_9N]^+$, $[C_3H_5NO]^+$	
	82-83	$[C_4H_6N_2]^+$, $[C_4H_5NO]^+$	

Supplementary Table 2. Potential fragment ions observed for the CDA mass spectra characteristic of the functional groups identified in this work and the associated CDA event identifiers, based on Postberg et al.[7]

References

89 Lee, N. *et al.* Measurements of freely-expanding plasma from hypervelocity impacts. *International Journal of Impact Engineering* **44**, 40-49 https://doi.org/10.1016/j.ijimpeng.2012.01.002 (2012).

90 Nouzák, L. *et al.* Laboratory measurements of the expansion characteristics of dust impact plasmas. *Icarus* **435** https://doi.org/10.1016/j.icarus.2025.116586 (2025).
